# Decommissioning in a local healthcare system in Sweden: responses to fiscal stress

**DOI:** 10.1186/s12913-020-05328-w

**Published:** 2020-06-03

**Authors:** Linda Moberg, Mio Fredriksson

**Affiliations:** 1 Department of Public Health and Caring Sciences, Health Services Research, Uppsala University, Box 564, 751 22 Uppsala, Sweden; 2 Department of Government, Uppsala University, Box 514, 751 20 Uppsala, Sweden

**Keywords:** Healthcare services, Decommissioning, Cut back management, Fiscal stress, Resource allocation

## Abstract

**Background:**

Drawing on the literature on cutback management, this article deals with healthcare decommissioning in times of austerity. Politicians and decision-makers are typically reluctant to decommission healthcare, and if they do, the public generally reacts strongly towards reductions in service supply. Despite this, comprehensive decommissioning does take place, though empirical knowledge about its effects and economic sustainability is limited. To further the understanding of healthcare decommissioning, this paper aims to introduce the concepts of cutback management into the research on healthcare decommissioning, and apply its components to an empirical case of comprehensive decommissioning. In doing so, the study analyses whether decommissioning measures can be expected to generate long- or short-term economic payoff, and considers what other effects they might have on the healthcare system.

**Method:**

We developed a theoretical framework that enabled us to investigate the measures through which a local healthcare system in Sweden, region Dalarna, responded to an acute fiscal crisis in 2014, and what effects these measures are likely to generate. The method used was a deductive content analysis of Dalarna’s decommissioning program, containing 122 austerity measures for saving 700 million Swedish Krona (SEK).

**Results:**

Dalarna’s local decision-makers responded to the fiscal crisis through a combination of operational cuts (20% of undertaken measures), programme cuts (42% of undertaken measures), and structural reforms (38% of undertaken measures). The instruments most commonly used were increased patient fees and the merger of service facilities. By relying foremost on programme cuts and structural reforms, Dalarna adopted the measures most plausible to have moderate or long-term economic payoffs. Successful implementation, however, may be challenging and difficult to evaluate.

**Conclusions:**

Healthcare politicians and decision makers have better potential to stabilize their long-term economic situation if they rely on responses such as operational cuts, programme cuts and structural reforms, as opposed to across-the-board cuts and cuts in investment and capital expenditures. However, with economics being only one important factor for sustainable healthcare systems, further studies should investigate how these measures affect important principles, such as equal healthcare distribution and access.

**Trial registration:**

Not applicable.

## Background

Drawing on the literature on cutback management, this article addresses healthcare decommissioning in times of austerity. Decommissioning is the planned process of removing, reducing or replacing healthcare services at the organisational level, and strategic reconfiguration of services that lead to organisational downgrading or closure. Decommissioning generally refers to an explicit approach in which the rationale and aims of decisions are made clear to all those involved, but may be passive through political neglect or less explicit through processes such as organisational mergers and takeovers not presented as decommissioning [1–3]. The focus in this paper is active decommissioning, which may include activities such as the termination of services, closure of specific providers, or reinvestment in cheaper alternatives [3, 4].

Decommissioning can occur at all levels in the healthcare system, and goes beyond prioritization between different services and patient groups which regularly take place in the healthcare system [5–7]. Active decommissioning is typically challenging for healthcare decision-makers since the public and important stakeholders within the healthcare organization, such as managers and clinicians, usually react strongly to it [5, 8, 9]. For the users of services, the negative effects of decommissioning such as impaired access and service quality, are often perceived as immediate and concentrated while the potential benefits are more diffuse and long-term, making such policies unpopular [10].

Although most actors within the healthcare system want to avoid active decommissioning, it does take place. Harlock et al. [3] alert us to healthcare decision-makers being increasingly faced with austere resources which force the issue of decommission, for instance by the withdrawal of resources from currently funded services. Due to the unpopularity of these policies, decision-makers often postpone these issues until quick decision-making and ad hoc rationing of services is required [11, 12]. Such circumstances make it difficult to make a marginal analysis of benefits and to thoroughly investigate the risks and economic consequences of specific decommissioning measures, implying that decision-makers seldom know with certainty whether their actions will generate the anticipated savings or what other effects they might have on the healthcare system [12].

The limited knowledge about the effects of decommissioning has been emphasized in previous research, suggesting that the contextual factors surrounding the enactment and implementation of decommissioning policies has not been the subject of sustained academic debate [2]. According to Williams et al. [1], this has entailed a lack of theoretical insights about how decommissioning can be designed and implemented. A crucial first step towards improving decommissioning theory and practice is thus to better understand how decommissioning programs empirically unfold in health systems. Without such knowledge, there is a risk that that blunt and unsophisticated instruments are employed, leading to unnecessary turmoil with no guarantee of positive outcomes [1].

This paper contributes to the filling of this empirical and theoretical gap by drawing on the literature on cutback management, which has developed to describe cutbacks in the public sector more broadly, but has not been systematically applied to healthcare. It offers different responses and instruments that national and local governments can adopt in austere financial climates, including wage freezes, program termination, increased user fees, and outsourcing of services [13]. Each response is associated with different drawbacks and merits, for instance whether they are most likely to have long-term or short-term payoffs with regard to economic stability (see [14] for an overview of this literature). Although it is well recognized in the literature on cutback management that a national or local government’s response to austerity and fiscal stress has major implications for the scope of their financial situation, few scholars within the field of healthcare governance have investigated what kinds of cutback measures healthcare decision-makers actually use when actively decommissioning services. Decommissioning studies have moreover typically focused on changes that occur on the clinical level in specific service areas, such as paediatric burn care services or end-of-life home support services [1], rather than comprehensive decommissioning programs that include many different responses to financial austerity within healthcare systems. In this study, we provide a novel contribution to the literature on decommissioning by investigating a comprehensive program which was a rapid and explicit response to a financially unsustainable situation in a local healthcare system in Sweden: region Dalarna. Due to longstanding budget deficits, Dalarna faced a major financial crisis in 2014, forcing the regional politicians to promptly enact a comprehensive decommissioning program including 122 decommissioning activities.

The aim of this study is twofold: To introduce the concepts of cutback management into the research on healthcare decommissioning, and to apply its components to an empirical case of comprehensive decommissioning. Through a case study of region Dalarna, the study analyses whether the undertaken measures can be expected to generate long- or short term economic payoff, and discusses what other effects they might have on the healthcare system in terms of access and quality. In doing so, the paper contributes to a deeper understanding of high-level decommissioning activities in healthcare systems. Such knowledge is relevant not only in the Swedish context, where many regions struggle to balance scarcity of resources with increasing demands for care [15]. The same challenges also face many healthcare systems in high-income countries such as the English National Health Service (NHS), where research suggests that substantial budget increases are needed in the coming years just to maintain the current level of service [16, 17]. For these countries, this paper offers a structured introduction to the approaches to crisis management presented in the cutback management literature, and how these approaches can be utilized when public healthcare systems are forced to implement decommissioning programs.

### Strategies for coping with fiscal stress

Since the publication of Levine’s seminal article ‘Organizational Decline and Cutback Management’ [18], scholars in the cutback management literature have studied how public organisations respond to fiscal stress. A plethora of strategies has been put forward, focusing foremost on the means through which governments can impose different kinds of spending cuts [14, 19–23]. In addition, it has also been suggested that governments can respond to economic downturns by implementing structural reforms that aim to improve the efficiency and organisational performance of public-sector organisations [20, 21, 24, 25].

Turning first to the discussion of how governments can cut public sector spending, the literature presents a basic distinction between ‘across-the-board’ and ‘targeted’ cuts. Across-the-board cuts, also referred to as ‘cheese slicing’, refer to measures where all sectors or divisions are urged to cut back by equal amounts or percentages [14, 21]. Conversely, targeted cuts imply that some divisions are faced with larger cuts than others and that these cuts are made on the basis of strategic prioritisation [14, 19, 26].

*Across-the-board cuts* can be seen as a rational response to fiscal stress since they reduce decision-making costs and are easy to implement without too much turbulence in the organization. Since all units are exposed to an ‘equal share of misery’, the cuts can be claimed to minimise the risks of intra-organisational conflicts [14, 18, 21, 22]. It has also been argued, however, that across-the-board cuts should be avoided since they are likely to reduce public-sector quality by penalising high-performing units and highly needed services to the same extent as those that are not as well-functioning [14, 19, 22, 27, 28]. Moreover, across-the-board cuts seldom correlate with a broader reform agenda through which governments with fiscal problems can facilitate economic stability in the long run [29]. Scholars have thus argued that across-the-board cuts might be appropriate for achieving short-term payoffs when the economic downturn is cyclical and not too deep. In more severe situations, however, targeted cuts are more appropriate for reducing public-sector spending [14, 19, 21, 23].

In general, there are three overarching types of targeted cuts which governments can adopt when faced with financial stress: cuts that aim to (i) reduce operational expenditures, (ii) reduce programme expenditures, and (iii) reduce capital and investment expenditures [14]. Through *operational cuts*, decision-makers typically aim to reduce their running expenditures in order to improve their budgetary situation on a short-term basis [14, 30]. Operational cuts are easy to implement and can be achieved either by reducing personnel costs or by cutting back on non-personnel expenditures [14]. When cutting personnel costs, the focus is mainly on limiting work hours and bringing down salary costs, which can be achieved by introducing wage freezes or stalling the rate of salary increases [14, 21, 30]. Other available instruments are to slow down the speed of promotion, reduce or control overtime, offer early retirement, fill positions with less credentialed and lower-paid staff, introduce hiring freezes, eliminate positions by adapting non-replacement policies, and laying off staff [14, 18, 23, 31, 32]. Among these instruments, hiring freezes and adoption of non-replacement policies have been emphasised as convenient short-term strategies for governments that wish to relieve their economic pressures in order to buy time and preserve their options. Moreover, they are perceived as comparatively painless instruments since they avoid redundancies and dismissals [14, 33]. Instruments such as layoffs and early retirements might also have the initial potential to rapidly reduce costs. In a more long-term perspective, however, it might be difficult to foresee whether these instruments would lead to any relevant knowledge and critical skills being lost to the organisation [14, 30]. In addition to cutting personnel costs, operational expenditures can also be reduced by cutting non-personnel costs, e.g., by restricting or banning spending on utilities, supplies, equipment, travel, conferences and complementary training [23, 32, 34].

The second strategy for targeted cuts suggests that governments can respond to fiscal stress by *cutting programme expenditures*. To do so, decision-makers typically need to reduce the adequacy of public services and benefits [14]. The instruments for cutting programme costs often vary among different sectors, but in general they tend to limit costs by reducing quality levels or by making it more difficult for citizens to access public services [14, 33]. This can for example be done by standardising the form and content of public services, reducing the requirements for service provision, reducing the variety of service tasks, limiting service hours, or terminating programmes. Additionally, governments can limit program costs by closing service facilities, increasing wait times, reducing transfers and public-entitlement levels, shortening reception times, or by shifting parts of the entitlement costs to the citizens, e.g., by establishing item charges and introducing or increasing user fees for services [14, 28, 33]. In addition, programme costs can also be cut through substitution, which refers to processes in which a specific treatment or method is replaced with one that is considered more efficient [4]. As long as the substituted treatment or method is more effective than the previous one, this kind of instrument can be adopted without compromising prevailing quality levels. In general, public organisations usually apply programme cuts to overcome economic pressure that threatens the organisation’s long-term survival [20]. Programme cuts are arguably more difficult to implement compared to operational and across-the-board cuts because they require some kind of organisational redesign and an analysis of which areas should be affected by the cuts and to what extent. Thus, the response typically inhibits quick reductions in spending but once the cuts have been implemented, it is likely that expenditures will be reduced and that there will be more moderate or long-term payoffs [30].

The last strategy for targeted cuts suggests that governments can respond to fiscal stress by *cutting investment and capital expenditures*. In order to reduce investment and capital expenditures, decision-makers apply different kinds of cancellation or postponement instruments, such as spending freezes for new capital projects, deferral of nonessential capital projects, and deferral of maintenance of facilities and equipment [14, 23]. Cancelling or postponing capital investments and maintenance are easily implemented when decision-makers want to achieve a short-term economic payoff. From a long-term perspective, however, Raudla et al. [14] warn that the costs are likely to increase over time. If such a cost increase correlates with a continued decline in resources, the subsequent costs for the cancelled maintenance might increase in an unpredictable way [14, 34].

In addition to across-the-board and targeted cuts, more resent research has argued that governments can respond to fiscal stress by *implementing structural reforms *that increase efficiency and organisational performance in the long run [20, 21, 24, 30, 35, 36]. According to Pollitt [21], structural reforms often appear to be the politically most desirable way to reduce costs because they allow politicians to claim that savings are possible while maintaining, or perhaps even improving, service quality (allowing them to ‘do more with less’). Hence, the political objective with this type of response is to implement managerial and organisational reforms that create more long-term savings but without having to compromise service quality [21]. Different types of reforms with this aim has been identified, e.g. to optimise old-, and introduce new work methods, mordernise workplaces, merge or centralise administrative tasks and public-service facilities (without laying off staff), digitalise public services, and outsource public tasks [20]. According to Pollitt, efficiency savings are important but might be risky because they demand organisational change and upheaval. As such, they are typically time-consuming and challenging to implement. Additionally, their results are often difficult to predict, especially if their aim and implementation strategy is vaguely formulated by the decision-makers. This means that structural reforms are often associated with uncertainty as to whether they will actually create the efficiency gains necessary to maintain prevailing quality standards while at the same time reduce the levels of public spending [21]. However, if the reforms turn out to be successful, they have the potential to restructure the organisation in such a way that its performance and economic situation can be improved in the long run [21, 24, 35].

Taken together, this overview suggests five overarching types of responses that governments can undertake when faced with fiscal stress. They can (i) cut expenditures across the board; (ii) cut operational expenditures; (iii) cut programme expenditures; (iv) cut capital and investment expenditures; or (v) use reforms to improve efficiency and organisational performance. The responses differ with regards to how easy or difficult they are to implement, how they create conditions for short- or long-term economic payoffs, and are associated with different plausible risks for the quality and function of the public sector (see Table 1).

**Table 1:** Possible responses to fiscal stress

Responses to fiscal stress	Implementation effort	Economic pay-off	Plausible risks
Across-the-board cuts	Easy	Short-term payoff	Long-term quality reduction by penalizing well-functioning and high quality units
Operational cuts	Easy	Short-term payoff	Long-term loss of important knowledge and skills
Programme cuts	Moderate	Moderate or long-term payoff	Quality reduction and/or impaired access
Capital expenditures and investment cuts	Easy	Short-term payoff	Long-term cost increase due to cancelled maintenance
Structural reforms	Difficult	Potential long-term payoff	Effects difficult to predict. May not lead to desired outcome, or any outcome at all

### The case: austerity and decommissioning in region Dalarna

In this paper, we conducted a case study in one of Sweden’s 21 regions: region Dalarna. While Sweden is often portrayed as an NHS system based on general taxation, universal rights, and strong state influence, it is in fact heavily decentralized. The responsibility for funding and provision of services lays primarily with the 21 politically governed and democratically elected regions, which levy proportional income taxes on their populations. In total, local income taxes fund approximately 75% of the healthcare services, with the rest coming from state subsidies and out-of-pocket payments.

Region Dalarna is the size of Belgium, but has only about 285,000 inhabitants (10 inhabitants per square kilometre). Compared to other Swedish regions, the population is less educated, is older, and assesses their health as being below the national average. There are about 30 primary care centres and six hospitals in Dalarna. The region employs approximately 8500 people and in 2017 its budget comprised approximately 8.2 billion SEK (~ 860 million euro). In 2014, Dalarna faced a major fiscal crisis. The outbreak of the crisis was preceded by 19 years of budgetary deficits. Attempts since 2010 to overcome the financial problems included, for instance, tax increases, a write-off the region’s deficit (which is contrary to the rules on good financial management per the Local Government Act, SFS 2019:835), the reduction of temporary staff, and the forcing of all healthcare divisions to cut their costs according to the cheese-slicing method identified above. These measures were however not sufficient to stop the economic downturn, and the prognosis for 2015 was a deficit of 320 million SEK. Due to the large budget deficit, the political leadership launched an urgent investigation to propose a detailed decommissioning program for how to save 700 million SEK (~ 70 million euro) between 2015 and 2019.

The politicians justified the cuts by stating that they were necessary for continued healthcare provision in the region, otherwise they anticipated a development where Dalarna no longer would be able to provide specialist healthcare to its citizens [11]. To increase the intra-organisational legitimacy of this process, public officials developed the decommissioning program in close collaboration with Dalarna’s four healthcare divisions and their operational managers. It consisted of two parts, which together comprised 122 measures through which Dalarna should reduce its healthcare costs, e.g. the closure of an ambulance station, the relocation of satellite primary care centres and specialist services from rural to urban areas, a reduced number of hospital beds, a freeze on hiring nurses and physicians, and increased out-of-pocket payments. In 2015, the political assembly approved the decommissioning program. By the end of 2016, implementation had been initiated for all but about 10% of the measures, and region Dalarna had saved approximately 300 million SEK [37].

## Methods

The aim with this paper is to introduce the concept of cutback management into the research on decommissioning and to apply its components to region Dalarna’s comprehensive decommissioning program. To reach this aim, the paper used deductive content analysis, a method based on a systematic classification of themes and structures in texts. In contrast to an inductive approach, which is used when no previous theory deals with the phenomenon under study, deductive content analysis is suitable when the general aim is to test or apply a previous theory in a new context [38]. In deductive content analysis, the structure of the analysis is thus operationalized based on previous research, and the research process typically begins with identifying key concepts or analytical categories. Thereafter, operational definitions for each category are determined [38].

To analyse what decommission measures Dalarna adopted, and what effects these might have on the region’s economic situation and healthcare system, we constructed an analytical framework based on the five different responses to fiscal stress identified in the literature on cutback management (see Table 2). The theoretical framework was then used to classify our analytical units, i.e., the austerity measures in Dalarna’s decommissioning program. In this process, we were open to the possibility that the region could have adopted other instruments than those specified in our analytical framework. On the few occasions that this occurred, we used the insights from previous research on cutback management to decide which overarching response the instruments represented.

**Table 2:** Analytical framework

Responses to fiscal stress	Examples of saving instruments	Plausible effects
Across-the-board cuts	All divisions are cut back in equal amounts or percentages	Short-term economic payoff and easy to implement. Might create long-term quality reduction by penalizing well-functioning and high quality units.
Operational cuts	*Personnel costs* Reduce or control overtime Salary cuts, e.g., wage freeze or stalled salary increases Slowdown of promotions Early retirement Fill positions with less credentialed or lower-paid staff Hiring freezes Eliminating positions Layoffs *Non-personnel costs* Restrict or ban spending on supplies, utilities and equipment Restrict or ban spending on travels, conferences and complementary training	Short-term economic payoff and easy to implement. Might create long-term loss of important knowledge and skills.
Programme cuts	Reduce quality requirements for healthcare provision Reduce variety of service tasks Standardise service forms and content Increase wait times Reduce service supply, e.g., by terminating programs, limiting service hours, closing service facilities or units Reduce public entitlements levels Limit reception time Introduce or increase user fees such as item charges, transport costs or fees for service Substitution of services or treatmens to reduce costs	Moderate or long-term economic payoff and moderate effort to implement. Might create quality reductions and impaired access.
Capital expenditures and investments cuts	Spending freezes for new capital projects and investments Deferral of non-essential capital projects and investments Deferral of maintenance of facilities and equipment	Short-term economic payoff and easy to implement. Might create long-term cost increase due to cancelled maintenance.
Structural reforms	Optimze old and introduce new work methods or work processes Merge service facilities or units (without laying off staff) Merge or centralize administrative tasks (whitout laying off staff) Digitilize public services New governance structures, such as outsourcing of public tasks	Potentially long-term economic payoff but difficult to implement. The effects are typically difficult to predict and the reforms may not lead to desired outcome.

The austerity measures were listed in the regional assembly’s public meeting agenda, containing all decisions politicians are to decide on during a given meeting. This list, however, only contained brief information about the austerity measures. To ensure that we understood the intentions behind the different measures and coded them correctly, we examined the preparatory work and outcome analyses, which formed the basis for the regional assembly’s decisions. Through these documents, which were attached to the meeting agenda, it was possible to categorise what responses Dalarna undertook and through which instruments the cuts and savings were to be realised. For instance, when the region decided to not replace retired nurses and to cap the allowed number of physicians to be hired, the measures were coded as operational cuts enabled thorough eliminated positions and hiring freezes. Similarly, the decision to reduce the number of hospital beds at the geriatric clinic in Ludvika was coded as a program cut by a reduction in service supply, and the decision to centralize the gynaecologist’s reception in Avesta to more urban areas was coded as a structural reform enabled through a merger of units.

The decommissioning program analysed in the study contained 122 austerity measures, through which Dalarna aimed to reduce their costs. Among these measures, six did not concern the healthcare system or its administration and eight did not propose any specific saving instrument, but rather explained the meaning of other austerity measures, for instance, with what frequency they should be evaluated. Thus, we have excluded these 14 measures from the analysis. An additional 18 austerity measures were not possible to categorise. For these measures, the characterisation was hindered either because we lacked sufficient information to determine how Dalarna expected the cost savings to be achieved, or because they only initiated further investigations, for instance the future political prioritizations in Dalarna’s healthcare system, and the future task description and structure of the region’s hospitals. It is plausible that the aim of these investigations was to propose structural reforms for increased efficiency and organizational performance. This was not possible to deduce from the region’s preparatory work and outcome analyses however, as the documents did not present what responses and saving instruments these investigations would actually result in. Thus, these austerity measures have also been excluded from the analysis. Taken together, the 32 measures excluded from the study were expected to save Dalarna approximately 345.5 million SEK (of which 330 million SEK was ascribed to the forthcoming investigation of Dalarnas future task description and structure of the region’s hospitals).

Finally, the decommissioning program also included four austerity measures where several saving instruments were merged together. For instance, when the region decided to raise the patient fees, it actually affected the fees in ten different healthcare areas. An additional four measures entailed more than one response to fiscal stress. E.g., a decision to terminate a service program also meant that Dalarna would lay off staff members, implying that it would lead to a programme cut as well as an operational cut. In order to ensure that we would not overestimate or underestimate a certain response or saving instrument, we treated each embedded response and saving instrument as a septate unit of analysis. After these adjustments, 118 individual austerity measures were included in the analysis.

## Results

### What decisions are made and how might they affect Dalarna’s economic situation and healthcare system

The categorisation of the austerity measures undertaken by Dalarna showed that the region did not respond to the fiscal crisis either through across-the-board cuts or by cutting investment and capital expenditures. Instead, Dalarna aimed to balance its healthcare budget by cutting operational and programme expenditures, and by improving its performance and efficiency through structural reforms. In the following section, these responses to Dalarnas austere financial situation will be presented more thoroughly.

### Operational cuts

Of the 118 austerity measures that Dalarna enacted, 20% can be categorised as operational cuts, meaning that the region aimed at reducing their running expenditures. Among these, 17% aimed to reduce non-personnel costs, whereas the remaining 83% aimed to cut personnel-related expenditures. Together, the operational cuts were expected to save at least 46.5 million SEK.

In order to reduce non-personnel-related costs, Dalarna decided to restrict spending on travels, external conferences and further training. These restrictions would foremost apply to the region’s politicians and central administrations (including the healthcare administration), but it was not specified what cost reductions they were expected to achieve. Moreover, Dalarna aimed to reduce their costs by spending less on healthcare facilities, in this case by reducing the space needed for a specific x-ray machine, and by terminating their previous support function, which was to assist the municipalities within the region in their work to improve public health (see Table 3).

**Table 3:** Instruments used to reduce operational costs

Frequency of instruments used for reducing operational costs	Expected (aggregate) savings, presented in million SEK
*Personnel costs*	
Eliminating positions, used six times	7.5 Specified for five of six instruments
Fill positions with less credentialed or lower-paid staff, used three times	3.9 Specified for one of three instruments
Hiring freeze (of hired physicians), used three times	27.5
Reduce or control overtime, used three times	1.6 Specified for two of three instruments
Layoffs, used three times	2
Impede performance of nonwork-related tasks during paid work hours, used once	Not specified
	**Total 42.5**
*Non-personnel costs*	
Restrict spending on travels, conferences and complementary training, used twice.	Not specified
Terminate support functions towards other organisations, used once	3.8
Reduce cost for facilities (reduced space), used once	0.2
	**Total 4**

In addition to cutting non-personal costs, Dalarna also sought to reduce its running expenditures by cutting personal cuts. The most frequently used instrument in this regard was to eliminate positions, which was addressed six times. In five of these cases, the region eliminated only one or a few positions, typically by not refilling them when members of the staff retired. Through the sixth measure, however, Dalarna wanted to eliminate positions corresponding to 600 employees by 2019. The expected savings of this specific measure, however, was not specified, and in 2016 Dalarna withdrew it without adjusting their overarching objective of saving 700 million SEK.

In addition, Dalarna intended to reduce personnel-related costs by laying off staff members, reducing or controlling overtime, and by filling positions with less credentialed or lower paid staff, typically by reducing the number of physicians and turning healthcare centres into nursing receptions. Moreover, they decided to investigate the possibility of halting the remuneration for union work, implying that employees should have less ability to perform non-work-related tasks during paid work hours. The expected saving of these instruments were relatively modest (see Table 3), and with regard to laying off staff, for example, Dalarna argued that the instrument only would affect a few staff members, typically when specific services or programmes were closed or terminated. Instead, Dalarna primarily tried to reduce their personnel costs by freezing the employment of hired physicians in primary care, psychiatric care and surgical care (Table 3). Based on the literature on cutback management, it can be seen as positive that Dalarna foremost aimed to eliminate positions and adopt hiring freezes. In addition to layoffs, these measures enable decision-makers to foresee what knowledge and competencies that risk being lost to the organisation in the long run.

### Programme cuts

The second way in which Dalarna responded to the financial crisis was by cutting programme expenditures. Of the 118 responses analysed in this study, 42% could be categorised as programme cuts. Hence, programme cuts are the most commonly adopted measure in the decommissioning program, and it is also the method through which Dalarna expected to gain the greatest savings, approximately 166 million SEK until 2019.

When programme expenditures are cut, it typically implies that decision-makers reduce the adequacy of public services, either by reducing the quality of the services, or by making it harder for citizens to access them. The most frequent way in which Dalarna attempted to reduce programme expenditures was by increasing patient fees for service and for medical transports, such as ambulance transports and need-based transport services to healthcare centres and hospitals. In total, this instrument was expected to save the region approximately 45 million SEK (see Table 4). In addition, Dalarna also adopted various instruments to reduce the supply of care. For instance, they closed three hospital units and terminated seven service programs, including a special pedagogical unit for children with multifunctional disabilities and a recreation service for people with disabilities. In addition, Dalarna also aimed to reduce the supply of care by limiting service hours, for instance by closing specific healthcare centres during the summer and by reducing the ordinary opening hours at a few hospital units. Furthermore, Dalarna also cut programme costs by reducing the production of care in order to match current staffing levels, and by reducing the variety of service tasks and by standardising the supply of services.

**Table 4:** Instruments used to reduce programme costs

Frequency of instruments used for reducing programme costs	Expected (aggregate) savings, presented in million SEK
Introduce or increase user fees for service and transports, used 18 times	44.6 Specified for 17 of 18 instruments
Limit service hours, used eight times	18.6
Perform less to match current staffing levels, used seven times	47
Terminate programmes, used seven times	25.6
Close service units and facilities, used three times	23
Reduce variety of service tasks or standardised service forms and content of service tasks, used three times	1.8
Substitution of services or treatment to reduce costs, used four times	5.2
	**Total 165.8**

Taken together, this implies that Dalarna cut program costs both by making it more difficult for inhabitants to access care (increasing user fees and reducing the supply of care) and by reducing the adequacy and quality levels of its services (reducing the supply and variety of services). Through four of the measures adopted in the decommissioning program, however, Dalarna attempted to reduce its programme costs through substitution, typically by replacing specific medicines with less costly alternatives. As long as the substitutes are not less effective than the previous treatment, this kind of programme cut can be made without compromising quality. Among the instruments used by Dalarna to make programme cuts however, only 3% of the proposed savings are expected through substitutions, with the remaining 97% of savings expected through increased patient fees and reductions in healthcare supply and variety.

### Structural reform to enhance performance and efficiency

In addition to operational and programme cuts, Dalarna also responded to the fiscal crisis by adopting reforms through which they hoped to increase the performance and efficiency of their healthcare system. Of the 118 analysed austerity measures, 38% could be categorised as proposals for structural reform, through which Dalarna expected to save a total of 104 million SEK.

Dalarna foremost aimed to increase the efficiency of their healthcare system by merging healthcare centres and hospital units. This instrument was addressed 27 times and expected to save the region approximately 53 million SEK (see Table 5). Merging two healthcare centres or hospital units can be seen as a structural reform for enhanced efficiency since the instrument implies that the same amount of healthcare services can be provided as prior to the merger, but that they will be centralised to fewer locations. This kind of centralisation was, according to Dalarna, expected to make it easier to staff the facilities and maintain good quality. Of the 27 mergers proposed by Dalarnas, however, 18 involved healthcare and specialist centres in more rural parts of the region merging with healthcare centres in the bigger cities, meaning that the provision of care was centralised to more densely populated areas of the region. Although the same amount of healthcare can still be provided in the region as a whole, this kind of measure can of course be perceived as a reduction of services in more rural areas, implying that the people living there might experience at least a perceived deterioration of access.

**Table 5:** Instruments used to create structural reforms

Frequency of instruments used for creating structural reforms	Expected (aggregate) savings, presented in million SEK
Merge service facilities and units, used 27 times	52.5
Optimise old or introduce new work methods and work processes, used 12 times	35.9 Specified for six of 12 instruments
Digitalisation of public services, used three times	0.6 Specified for one of three instruments
Merge and/or centralise administrative tasks, used twice	15 Specified for one of two instruments
New governance structures, used once	Not specified
	**Total 104**

In addition to merging service facilities and units, Dalarna also used structural reforms in order to optimise old or introduce new work methods and processes. This instrument was employed 12 times, and suggested, for example, that Dalarna should restructure their prosthetic surgery so that fewer patients would have to go to other regions for treatment. The savings expected from altering working methods and processes were only presented for six of the 12 instruments. Moreover, it is only with regard to 7 of these instruments that Dalarna specified the new methods and processes through which the staff was expected to work. For the remaining five, the region merely decided that the possibility of introducing new methods should be further investigated, e.g. whether it would be possible to introduce a mobile team of nurses that could visit the more rural areas in the region. Whether these instruments actually will be implemented, and what savings they can give rise to is thus difficult to predict.

Dalarna further aimed to reduce their spending by centralising administrative tasks, and by investigating the possibility of digitalising certain healthcare services. Finally, Dalarna aimed to adjust its governance structure by introducing a new model for planning and steering healthcare services (*planerings- och styrningsmodell*), which should enable the region to focus more on results and medical outcomes when planning, monitoring and evaluating the healthcare system. However, it was not specified what savings the new model was expected to generate.

Taken together, the literature on cutback management suggests that structural reforms typically are time-consuming to implement and that their outcomes are difficult to anticipate, especially if the aim of the reform is vaguely expressed [23]. For Dalarna, this implies that the ambition to merge healthcare facilities and units might be a rather straightforward process. What effect, if any, the reforms on new working methods and digitalization might have on the healthcare system is, however, less certain [23].

## Discussion

To contribute to the literature about healthcare decommissioning, and what potential effects different responses to fiscal stress in this context might have, this study aimed to introduce the concepts of cutback management into research on healthcare decommissioning, and apply its components to an empirical case of comprehensive and explicit decommissioning. The case consists of a local Swedish healthcare system (Region Dalarna) that enacted a wide-raging decommissioning program in 2015, with the aim to achieve a balanced budget. The case study was structured around five possible responses to fiscal stress: (i) across-the-board cuts, (ii) operational cuts, (iii) programme cuts, (iv) capital and investment cuts, and (v) structural reform.

The findings showed that Dalarna did not use across-the-board cuts, or cuts in investment and capital expenditures. Rather, the region used a targeted or strategic approach, relying on a combination of operational cuts (20%), programme cuts (42%), and structural reforms (38%). According to theory from cutback management, the non-use of across-the-board cuts should, in a long-term perspective, be favourable for the quality of healthcare services in Dalarna as ‘cheese slicing’ is likely to reduce overall quality since well-functioning services and services with a balanced economy are forced to make cuts as well [18, 19, 21]. Similarly, Dalarna abstained from making short-term savings through cuts in capital expenditures and investments which may increase long-term costs and have negative long-term effects on quality over time as the need for investment and maintenance is likely to increase over time [14, 34].

Among the responses that Dalarna undertook, it is commonly claimed that operational cuts have the most short-term economic impact [14, 30], but that the cuts can lead to unexpected consequences depending on the instruments used to achieve them. For instance, hiring freezes and eliminated positions, which amounted to 39% of operational cuts in Dalarna, are often advocated since they, in contrast to layoffs (13%), enable governments to foresee what knowledge and competencies that will be lost to the organisation in the long run [14, 30, 33]. Thus, the case study suggests that Dalarna’s operational cuts, at least in theory, created better conditions for controlling the future supply of competencies. This is highly important since lack of skilled staff is one of the Swedish health system’s greatest challenges and is expected to continue to be so also in the future [39].

According to the literature on cutback management, structural reform or program cuts, which amounted to 80% of the decommissioning activities in Dalarna, are the responses with the highest potential to enable long-term economic improvement [14, 20, 21]. This suggests that Dalarna adopted activities with potential to turn their public finances in a more sustainable direction. However, structural reforms are typically time-consuming to implement and the effects difficult to anticipate due to other simultaneous developments, suggesting that their potential long-term effects are often uncertain and difficult to evaluate. Moreover, since structural reforms primarily aim to achieve efficiency gains, decision makers can claim that savings will be made while quality is maintained or improved (gain without pain). As such, structural reforms sound less threatening than cuts and appear to be the politically most desirable way to handle financial turbulence [21]. Based on this, it could be asked whether this response is an example of decommissioning or an alternative to it. In our opinion, the literature on cutback management helps clarify this ambiguity by making visible that it depends on the aim and/or the outcome of the structural reform. If the aim is to reduce costs, or if it creates (unforeseen) reductions in service supply, it should be treated as an example of decommissioning. In the case of Dalarna, 60% of the responses categorized as structural reforms implied mergers of healthcare facilities and units, typically through centralization. The stated aim of the full program and the included structural reforms was to reduce the region’s healthcare costs and compared to more vaguely worded and future-oriented reforms, mergers are rather feasible to implement. For people living in rural areas affected by the mergers, e.g. in primary care or in women and children’s care however, it is plausible that the measures were perceived as a deterioration of access.

Furthermore, it is important to point out that programme cuts typically reduce the adequacy of public services such as healthcare, either by altering the quality goals and standards, or by making it more difficult for patients and citizens to access the services [14, 28, 33]. In total, 46 of the 50 measures through which Dalarna aimed to cut program cuts involved increased fees and reduction in healthcare supply and service variety. One such example was the planned closing of all heated rehabilitation pools. Although the program cuts could make Dalarna’s public finances more long-term sustainable, the adoption of such measures require a thorough evaluation of how the publics’ need and access to care develops over time. Otherwise, there is a risk that the publics’ access to care will deteriorate.

Naturally, the public may fear a number of potential negative consequences from decommissioning, such as reduced access and quality. This fear was also expressed by residents in the region of Dalarna [40]. They argued that access would be negatively impacted, particularly in rural areas where satellite primary care centres were closed, and that the quality of services would be negatively impacted, in particular for people with disabilities, leading to worse physical and mental health. Residents also pointed to potential unintended human consequences such as suicides, unemployment and unnecessary deaths from delayed care [40].

Following the reasoning by Henderson et al. [41] and Freeman et al. [42], the availability of services in Dalarna’s more rural areas has to some degree deteriorated as a result of the decommissioning program, since geographical accessibility has declined (e.g. with the mergers and the closure of satellite primary care centres). Access is, however, a complex concept. It has, for instance, been suggested that access in rural areas may in fact be related to car ownership, which negatively influences the poor, elderly, and women who are to a lesser extent car owners [43]. To some extent, digital- or telehealth solutions (which were proposed by Dalarna) can increase availability, although there are indications that the elderly are less satisfied with digital primary care as a compensation for face-to-face services [44, 45]. Generally, there is a lack of knowledge about what people in different areas or countries consider an acceptable level of local service provision. Data from 2017 indicates that a large proportion (72%) of the Swedish population is positive towards the concentration of hospital services if it entails an increase in quality. The share was even higher in Dalarna (about 76%) [46]. To what degree the population is positive towards the concentration of primary care services is unknown. However, it seems as if those protesting against the closings of the satellite primary care centres in Dalarna prioritized geographical access above higher quality and more continuous staffing at the health centres in the urban areas [40]. In line with this, previous research shows that a relationship between traveling further and having worse health outcomes cannot be ruled out [47], and that a reduction of primary care provision locally may negatively affect the creation of social capital and attenuate the identity of the community [48].

As a whole, this study suggests that the decision-makers in Dalarna relied on a combination of operational cuts, programme cuts, and structural reforms. Rather than reducing the supply of care, the decision-makers most commonly aimed to reduce spending by increasing patient fees and merging service facilities, typically by centralising the provision of care. According to theory on cutback management, these kinds of responses have the highest potential to enable moderate or long-term impacts on the local healthcare economy, but further studies are needed to investigate how these measures affect important system goals such as access and quality. A recent update on healthcare quality in Dalarna suggests an overall improvement in comparison to the other Swedish regions between 2014 and 2018, but with large variations between different types of services. In comparison to the other regions, access improved between 2014 and 2016, but dropped again 2018 and remains well below the national average [49]. Further investigation is needed to understand in what way, if at all, patterns of quality and access are related to the decommissioning programme that was adopted in 2015. Concerning quality, it is crucial to further investigate structure-, process- as well as outcome quality, distinctions that are not made in the cutback management literature.

In our opinion, the literature on cutback management may help both researchers and decision-makers to make visible the full range of potential responses to poor finances, which are not limited to changes to the *services* provided (e.g. withdrawal or reduction in service supply, restriction and substitution) [4]. Many other types of responses, which might take place ‘behind the scenes’ or be less obvious to the public or health professionals, may be adopted to cut healthcare organisation’s costs, such as reduction or control of overtime, adaption of non-replacement policies, restricted spending on travels, or postponed capital investments. With regard to the specific case of Dalarna, however, further research is necessary to investigate the economic effects, which today are inconclusive. An assessment conducted on behalf of the region’s auditors stated that about half of the expected savings has been achieved [50]. However, it proved difficult to track the realised savings for all the measures separately, implying that the calculations were based on aggregated data. Moreover, the region had no clear evaluation of their proposed structural reforms and action plans, making it difficult to estimate the financial impact of the decommissioning program going forward [51]. It is thus precarious to say whether the measures of short-term potential have generated the expected savings, and to what extent those with more long-term potential are likely to lead to the expected savings, or have led to savings so far. It is also not clear what definition of “long-term” is used when discussing healthcare budgets, and the longer the time perspective, the more difficult it becomes to track the effects of a particular cutback measure. Furthermore, it is important to acknowledge that the need for decommissioning can be calculated differently, depending for instance on which costs that are included [52]. This was reflected by the citizens who protested against Dalarnas decommissioning program, and questioned the decision makers’ calculations and stated savings [40]. According to protesters, the decommissioning program risked leading to so called unmanaged substitution [4], implying that the use of non-targeted services would increase and thus cause an overall increase in healthcare costs. Similarly, Robinson et al. [5] argued that decision-makers rarely consider the impact service closures will have on the demand for other services, and that these effects are also highly difficult to evaluate.

Finally, we acknowledge some limitations to this study. First, we have only been able to describe the measures decided on by region Dalarna in the decommissioning programme and we cannot within the scope of this article track the level of savings from the measures. This would be a next step to further investigate whether the responses to fiscal stress identified in the literature on cutback management have the anticipated economic effects when applied to healthcare decommissioning. Furthermore, we do not consider how this specific combination of measures in Dalarna came about. Although a previous study showed that the clinic managers and their staff was highly involved in developing the details of the decommissioning programme [11], more studies are needed to further the understanding about the nature of the decision-making process. Moreover, we suggest that further research apply a comparative perspective by investigating if and why responses to fiscal stress differ between healthcare contexts.

## Conclusions

Through this study, we interlink the research fields of healthcare decommissioning and cutback management. Decommissioning is an emergent field of research and by connecting it to cutback management this study illustrates the various responses available for governments that aim to reduce their public spending in order to manage fiscal stress and budgetary deficits. By applying the literature on cutback management to a case of comprehensive decommissioning (the Swedish region Dalarna), the study also adds to the understanding of how decommissioning programs unfold in healthcare systems, which according to Williams et al. is a crucial step towards improving decommissioning policy and practice [1]. Through the case study, we found that Dalarna’s decommissioning program contained a combination of operational cuts, programme cuts and structural reforms. By relying on these kinds of responses, rather than adopting across-the-board cuts and cuts in investment and capital expenditures, healthcare decision-makers have a better potential to stabilize the long-term economic situation. However, with economics being only one important factor for sustainable healthcare systems, further studies should investigate how such measures affect important principles, such as access and quality.

## Data Availability

The datasets generated and analysed during the current study are available from the corresponding author on reasonable request.

## Abbreviations

**NHS**: National Health Service
**SEK**: Swedish Krona
